# Supplementary material for: Proteomic Determination of Low-Molecular-Weight Glutenin Subunit Composition in Aroona Near-Isogenic Lines and Standard Wheat Cultivars
Source: Int J Mol Sci. 2021 Jul 19;22(14):7709. doi: 10.3390/ijms22147709 (PMC8306524; doi:10.3390/ijms22147709)
Supplement: Supplementary file 1 [file ijms-22-07709-s001.zip › ijms-1291691-supplementary.pdf]

Supplementary Material

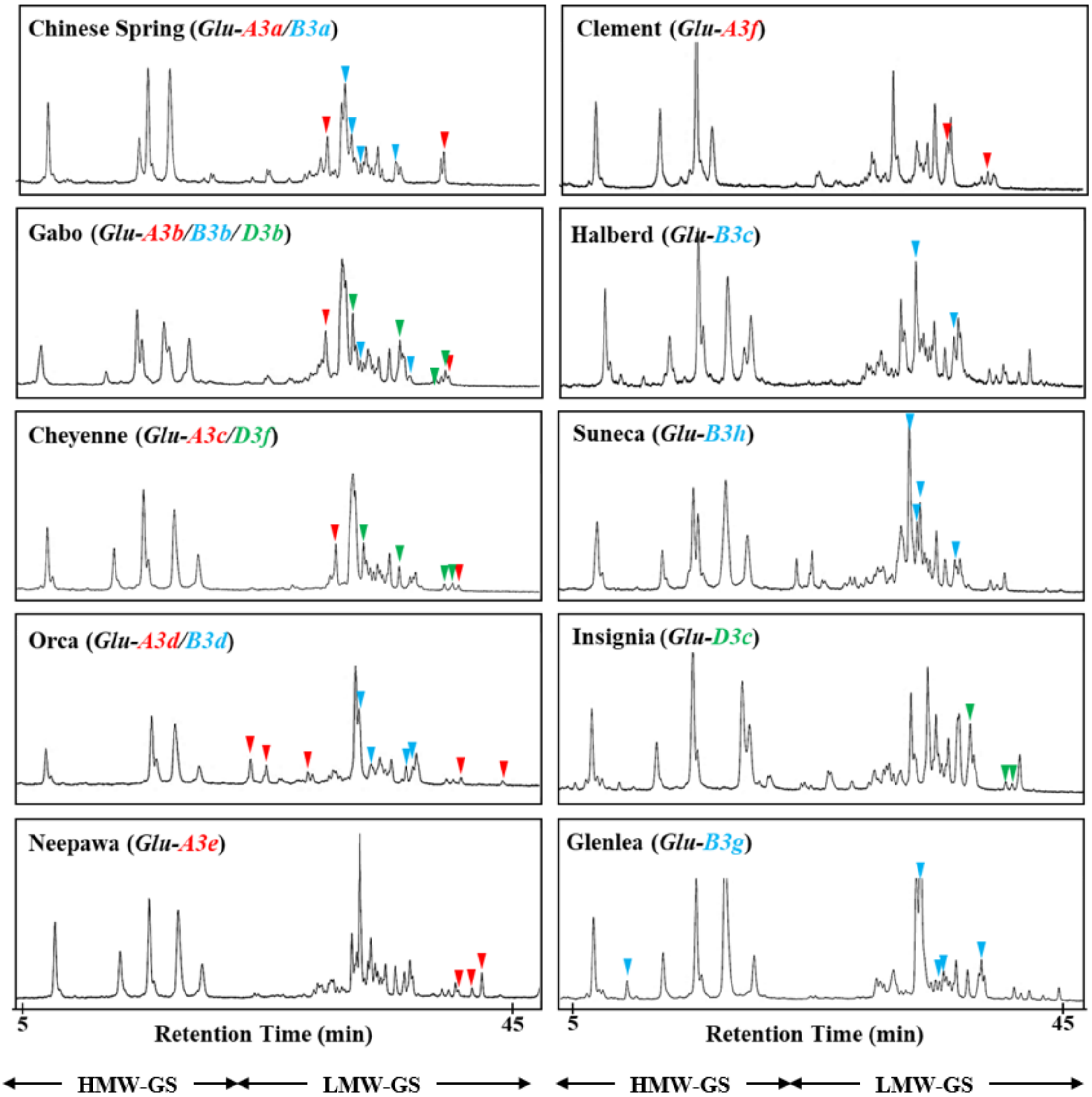

Figure S1. RP-HPLC analysis of LMW-GS fractions in 10 standard wheat cultivars, 'Chinese spring' (*Glu-A3a/B3a*), 'Gabo' (*Glu-A3b/B3b/D3b*), 'Cheyenne' (*Glu-A3c/D3f*), 'Neepawa' (*Glu-A3e*), 'Clement' (*Glu-A3f*), 'Halberd' (*Glu-B3c*), 'Orca' (*Glu-A3d/B3d*), 'Suneca' (*Glu-B3h*), 'Insignia' (*Glu-D3c*), and 'Glenlea' (*Glu-B3g*). Peaks corresponding to each LMW-GS allele encoded by *Glu-A3*, *Glu-B3*, and *Glu-C3* are indicated by red, light blue, and green arrowheads, respectively.

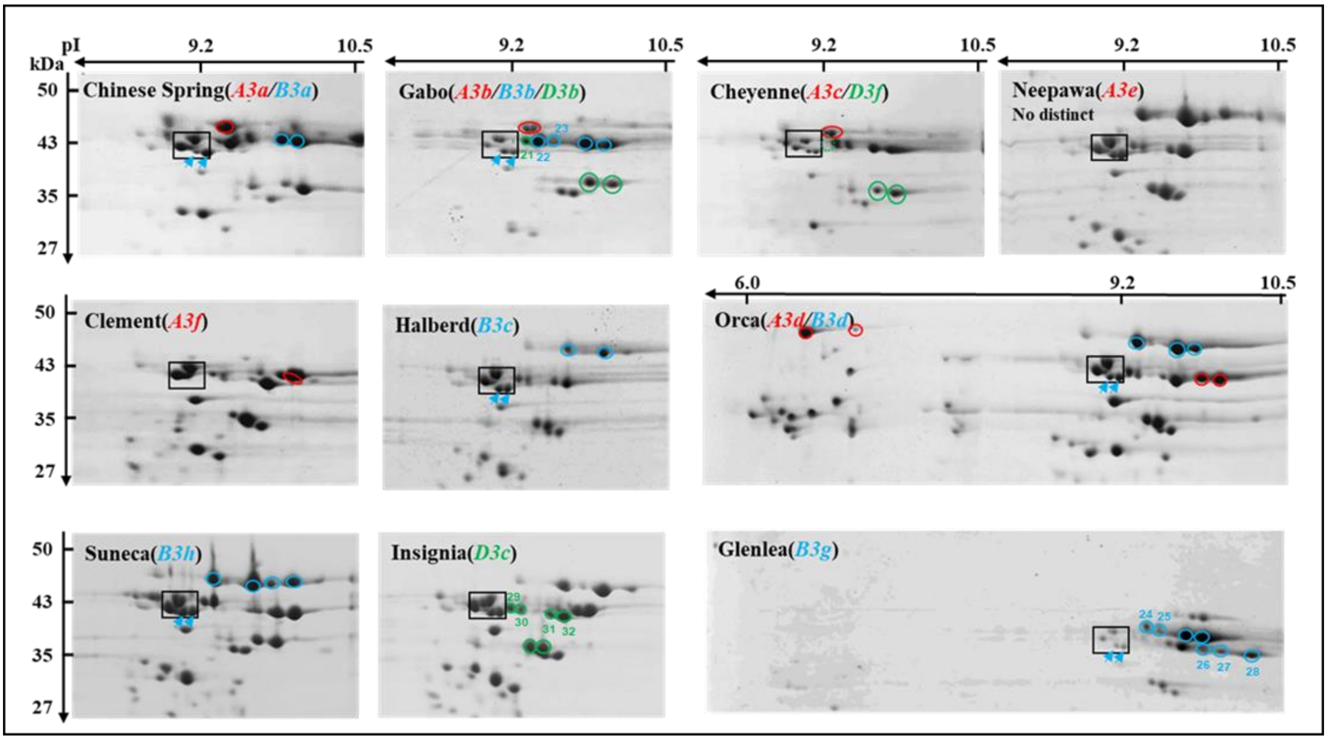

Figure S2. Two-DGE analysis of LMW-GSs from 10 standard wheat cultivars: 'Chinese spring' (*Glu-A3a/B3a*), 'Gabo' (*Glu-A3b/B3b/D3b*), 'Cheyenne' (*Glu-A3c/D3f*), 'Neepawa' (*Glu-A3e*), 'Clement' (*Glu-A3f*), 'Halberd' (*Glu-B3c*), 'Orca' (*Glu-A3d/B3d*), 'Suneca' (*Glu-B3h*), 'Insignia' (*Glu-D3c*), and 'Glenlea' (*Glu-B3g*). Protein spots corresponding to each LMW-GS allele encoded by *Glu-A3*, *Glu-B3*, and *Glu-C3* are indicated by red, light blue, and green arrows, respectively. Numbered spots were identified using LC–MS/MS.

Table S1. Standard wheat cultivars[a] used to distinguish alleles at the *Glu-A3, Glu-B3, Glu-D3* loci

Allele	Standard Cultivar
Glu-A3a	Chinese Spring
Glu-A3b	Gabo
Glu-A3c	Cheyenne
Glu-A3d	Orca
Glu-A3e	Neepawa
Glu-A3f	Clement
Glu-B3a	Chinese Spring
Glu-B3b	Gabo
Glu-B3c	Halberd
Glu-B3d	Orca
Glu-B3g	Glenlea
Glu-B3h	Suneca
Glu-D3b	Gabo
Glu-D3c	Insignia
Glu-D3f	Cheyenne

[a] Standard wheat cultivars are referenced by Lee *et al*.(2017)

Table S2. Identification of LMW-GSs at the *Glu-A3*, *Glu-B3* and *Glu-D3* loci in standard wheat cult ivars

# spot	Cultivars	Gene	MS/MS identification	[a]Gene haplotype	N-terminal sequence	# A.A	[b]Putative Corresponding Genes	
							Accession No. (identity)	Gene
21	Gabo	*Glu-D3b*	AEI00677	*GluD3-31*	MENSHIP	334	JX878006 (100%)	*D3-578b*
22	Gabo	*Glu-B3b*	ACA63875	*GluB3-22*	MENSHIP	349	EU369721 (100%)	*B3-621a*
23	Gabo	*Glu-B3b*	AFU48612	*GluB3-22*	MENSHIP	349	EU369721 (99%)	*B3-621a*
24	Glenlea	*Glu-B3g*	AWK59751	*GluB3-21*	MENSHIP	350	EU369704 (100%)	*B3-624*
25	Glenlea	*Glu-B3g*	AWK59751	*GluB3-21*	MENSHIP	350	EU369704 (100%)	*B3-624*
26	Glenlea	*Glu-B3g*	ABY58126	*GluB3-15*	MENSHIP	323	EU369703 (100%)	*B3-544*
27	Glenlea	*Glu-B3g*	ABY58126	*GluB3-15*	MENSHIP	323	EU369703 (100%)	*B3-544*
28	Glenlea	*Glu-B3g*	ABY58126	*GluB3-15*	MENSHIP	323	EU369703 (100%)	*B3-544*
29	Insignia	*Glu-D3c*	ABY58133	*GluD3-32*	IENSHIP	334	FJ755316 (99%)	*D3-578a*
30	Insignia	*Glu-D3c*	ABY58133	*GluD3-32*	IENSHIP	334	FJ755316 (99%)	*D3-578a*
31	Insignia	*Glu-D3c*	ABY58133	*GluD3-32*	IENSHIP	334	FJ755316 (99%)	*D3-578a*
32	Insignia	*Glu-D3c*	ABY58133	*GluD3-32*	IENSHIP	334	FJ755316 (99%)	*D3-578a*

[a] LMW-GS genes identified from *Glu-B3* (Wang *et al*., 2009) and *Glu-D3* (Zhao *et al*., 2007)

[b] LMW-GS genes isolated from Aroona and ARILs (Zhang *et al*., 2012 and 2013)
